# Effects of a low‐carbohydrate/high‐protein diet on metabolic health in individuals with chronic spinal cord injury: An exploratory analysis of results from a randomized controlled trial

**DOI:** 10.14814/phy2.15501

**Published:** 2022-11-21

**Authors:** Jia Li, Barbara Gower, Amie McLain, Ceren Yarar‐Fisher

**Affiliations:** ^1^ Departments of Physical Medicine and Rehabilitation The University of Alabama at Birmingham Birmingham Alabama USA; ^2^ Department of Nutrition Sciences UAB School of Health Professions Birmingham Alabama USA; ^3^ Departments of Physical Medicine and Rehabilitation and Neuroscience The Ohio State University Columbus Ohio USA

**Keywords:** metabolic health, nutrition, spinal cord injury

## Abstract

We explored the impact of a low‐carbohydrate/high‐protein diet (LC/HP, ~30% energy from protein, 40% energy from carbohydrate) on indices of metabolic function and body composition in individuals with chronic spinal cord injury (SCI). Adults with SCI (≥3 years post‐injury, C4‐L2, AIS A‐D) and insulin resistance or pre‐diabetes were randomly assigned to an 8‐week iso‐caloric LC/HP diet group (*n* = 11) or control group (*n* = 14). All LC/HP meals were delivered weekly to participants' homes, and participants in the control group consumed their habitual diet. Each participant underwent an oral glucose tolerance test (OGTT) to assess glucose tolerance, insulin, area under the curve (AUC) for glucose and insulin, Matsuda Index, glucose‐stimulated insulin secretion (GSIS), disposition index, and hepatic insulin extraction (HIE). Fasting blood lipid and inflammation were assessed, and body composition was estimated using dual‐energy x‐ray absorptiometry. A linear mixed model was used to evaluate the main effect of diet, time, and their interaction. Compared to the control group, participants in the LC/HP group had reduced total body fat mass (LC/HP: −5.9%, Control: 0.7%), visceral fat mass (LC/HP: −16.2%, Control: 5.2%), total‐ (LC/HP: −20.1, Control: 3.7 mg/dl), and LDL‐cholesterol (LC/HP: −13.9, Control: 3.1 mg/dl) (*p*_diet*time_ < 0.05 for all). Regardless of group, AUC_insulin_ and peak insulin during the OGTT decreased, and HIE increased over time (*p*_time_ < 0.05). A trend for diet*time interaction was observed for glucose_OGTT120min_ (LC/HP: −20.7, Control: 3.0 mg/dl, *p*_diet*time_ = 0.09) and peak C‐peptide (LC/HP: −2.1, Control: 0.0 ng/ml, *p*_diet*time_ = 0.07). HDL‐cholesterol, lean body mass, Matsuda Index, fasting glucose, insulin, insulin_OGTT120min_, AUC_glucose_, pancreatic beta cell function (GSIS, disposition index), and inflammation (C‐reactive protein, IL‐6, IL‐8, IL‐10, TNF‐α) did not change over time. In conclusion, our results suggest that individuals with SCI and insulin resistance may adopt an LC/HP diet to improve body composition and lipid profiles. Its impact on glucose metabolism and inflammation remains inconclusive and warrants future investigations.

## Graphical abstract

Our results support a favorable change of the body composition and blood lipid profile after consuming a healthy LC/HP diet for 8 weeks in individuals with chronic SCI. This underscored the importance of dietary modifications for metabolic health in SCI.

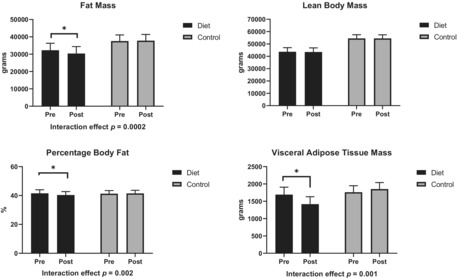

## 1 INTRODUCTION

With the advances in medical treatment, individuals with spinal cord injury (SCI) tend to live much longer today compared to past decades (National Spinal Cord Injury Statistical Center, 2020). However, as a result of SCI‐induced physiological, psychological, and lifestyle changes (Bauman & Spungen, 1994, 2000; Inskip et al., 2010; Nash et al., 2016), individuals with SCI are at an increased risk of developing secondary conditions, such as metabolic disorders and cardiovascular diseases (Bauman & Spungen, 1994; Jensen et al., 2012) as well as earlier age‐related functional declines (Hitzig et al., 2011). This is thought to partly result from SCI‐associated changes in autonomic function, body composition (e.g., severe muscle atrophy accompanied by increased adiposity), physical inactivity, and hormonal imbalance (Jensen et al., 2012; Krause & Saunders, 2011; Phillips et al., 1998). In particular, given that skeletal muscle is the main determinant of resting energy expenditure (Zurlo et al., 1990) and is responsible for taking up 70%–90% of glucose from the blood in the postprandial state (Evans et al., 2019), the severe muscle loss after SCI contributes to the energy imbalance and impaired glucose tolerance in this population. Furthermore, increased adiposity, particularly visceral adipose tissue, may further contribute to the development of chronic inflammation (Monteiro & Azevedo, 2010; Wang et al., 2007), as well as lipid and glucose metabolism disorders (Bauman & Spungen, 2001; Klop et al., 2013).

Dietary modification may be a key strategy for improving metabolic health in individuals with SCI. A few studies reported that they often consume low‐quality diets, characterized by a high intake of fat, added sugar, and low intake of fruits and vegetables (Groah et al., 2009; Li et al., 2021; Silveira et al., 2019). Such diets may further increase the risk of metabolic diseases (Morze et al., 2020; Nicklas et al., 2012). Results from a limited number of dietary research studies support that adopting a generally healthy diet (e.g., fruits and vegetables, whole grains, low added sugar) and lifestyle regimen can benefit weight control and metabolic health (Allison et al., 2017; Chen et al., 2006; Gorgey et al., 2012; Myers et al., 2012; Radomski et al., 2011; Sabour et al., 2018). However, none of these studies evaluated the efficacy of a specific dietary modification using a controlled feeding design in individuals with SCI.

To address this gap in the literature, we designed the current study based on the understanding of SCI‐induced physiological changes and the effects of a low‐carbohydrate/high‐protein diet (LC/HP) on several risk factors for metabolic disorders observed in non‐SCI populations (Conlon & Bird, 2014; Yu et al., 2016). A higher protein diet confers its benefits in several ways: (1) dietary protein has the highest thermic effect of feeding among the macronutrients, which improves energy balance for weight control purposes (Calcagno et al., 2019); (2) a meal higher in protein with concomitantly reduced carbohydrate content elicits lower postprandial glucose responses (Gannon & Nuttall, 2004; Nuttall & Gannon, 1991). Postprandial glucose response is not only the main determinant of the overall glucose control (i.e., hemoglobin A1c), but also an independent risk factor for the development of cardiovascular diseases (Mann et al., 2019; Monnier & Colette, 2006); and (3) a meal with higher protein and reduced carbohydrate content can improve blood lipid profiles by limiting substrate availability (i.e., carbohydrate) for de novo lipogenesis in the liver (Sanders & Griffin, 2016). Thus, an LC/HP diet may counteract some of the maladaptive physiological changes with SCI. In the current study, we hypothesized that an LC/HP diet (carbohydrate: ~40% energy, protein: ~30% energy, fat: ~30% energy) that includes healthy dietary components (e.g., lean meat, whole grains, fruits, and vegetables, etc.) could induce favorable adaptations in indicators of metabolic health (e.g., body composition, glucose tolerance, insulin resistance, blood lipid profile) and chronic inflammation among individuals with SCI.

## 2 METHODS

### 2.1 Study design

Data presented in the current study are part of a larger dataset. Data pertaining to the gut microbial changes to the dietary intervention was previously published elsewhere (Li et al., 2022). Eligible participants who provided informed consent were randomly assigned into an LC/HP diet group or a control group in a 1:1 ratio. Randomization was performed using the block randomization method (block size 4) by the study statistician. Assignments were placed into closed envelopes and given to each study participant by the study coordinator (Li et al., 2022). All nurses performing blood draws and core facilities analyzing the study outcomes were blinded to group assignment. Outcome measures were performed at two time points: at baseline and after completion of the 8‐week LC/HP diet. Post‐study assessments were performed on the morning after the last day of the intervention (Li et al., 2022). The study duration was chosen because it is commonly used in dietary intervention efficacy studies (Bozzetto et al., 2012; Bradley et al., 2009; Brooking et al., 2012; Lopez‐Legarrea et al., 2013; Tay et al., 2008; Te Morenga et al., 2011).

### 2.2 Eligibility criteria

**FIGURE 1:**
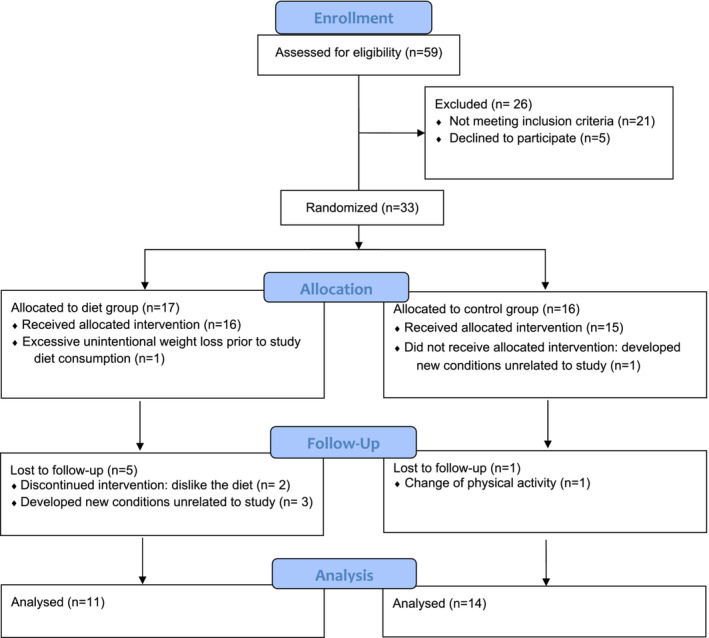
CONSORT flowchart

Participants were eligible if they: (1) were between the ages of 18 and 65 years; (2) had a traumatic SCI at the cervical, thoracic, or lumbar level (C5–L2) classified as American Spinal Injury Association impairment scale A, B, C, or D; (3) impaired glucose tolerance or insulin resistance; (4) no history of pre‐existing self‐reported type 2 diabetes, renal disease, or pressure injury; (5) were at least 3 years post‐injury (based on data collected from our existing studies, we have observed that glucose intolerance develops as soon as 3 years post‐injury among our participants); (6) had not participated in a weight loss program for the last 6 months; (7) had not habitually consumed a HP diet (~30% energy from dietary protein); (8) had not been on antibiotics for at least 4 weeks before the study, and (9) had not taken medications for weight management, blood glucose, and lipid disorders. Interested participants underwent an oral glucose tolerance test (OGTT) to confirm eligibility (Li et al., 2022). We complied with the American Diabetes Association recommendations (American Diabetes Association, 2017) to identify participants with impaired glucose tolerance (2‐h OGTT serum glucose 140–199 mg/dl). Participants with a Matsuda index below 4.3 were considered insulin resistant (Takahara et al., 2013). Eligible participants were excluded if they developed new health conditions (e.g., pressure injury, kidney disease) that would affect study outcomes or inhibit them from participating or started on new medications. A total of 59 participants underwent the screening procedures, and 33 eligible participants were assigned to either the LC/HP diet group (*n* = 17) or the control group (*n* = 16). In the diet group, one participant was excluded due to weight loss before starting the study diet, and five participants discontinued the study diet (three developed new conditions unrelated to the study diet, and two did not like the study diet). Among participants assigned to the control group, one developed a new condition unrelated to the study diet, and one was excluded due to starting new exercise regimens (Figure 1, Consort flow diagram).

### 2.3 LC/HP diet intervention group

Each participant's usual dietary intake was assessed by three 24‐h dietary recalls (2 non‐consecutive weekdays and 1 weekend day) administered by the research coordinator. Initial calorie prescription was estimated using the Harris‐Benedict equation, and an activity factor of 1.2 was used. The study dietitian planned personalized menus consistent with the LC/HP diet parameters (for the LC/HP diet group) (Li et al., 2022). During the first 2 weeks, participants tried a 6‐day‐rotating menu (i.e., menus were repeated every 6 days, includes breakfast, lunch, snack, and dinner) and decided if they wanted to change/substitute any food items or increase/decrease calorie prescription. Then the requested changes in the study menu was implemented at the beginning of the third week while ensuring the LC/HP diet parameter. The LC/HP diet parameters fall within the acceptable macronutrient distribution range established by the Institute of Medicine (2005). Dietary fat sources focused on monounsaturated and polyunsaturated fats, for example, plant oils and nuts; dietary carbohydrate sources emphasized whole grains, fruits, vegetables, and legumes; and dietary protein sources included lean meats, fish, chicken, eggs, and non‐fat dairy foods. Participants in the LC/HP were allowed to consume non‐caloric drinks ad libitum (e.g., water, diet drinks, and unsweetened tea/coffee without any cream). The study provided LC/HP meals and delivered them to participants' homes weekly.

### 2.4 Control group

Participants assigned to the control group did not receive any dietary intervention and continued with their regular diets. Participants' dietary intake was assessed using three, 24‐h dietary recalls (on two non‐consecutive weekdays and 1 day on the weekend), three times (at baseline, week 4, and 8) to confirm that there were no substantial changes in their diet during the study (Li et al., 2022). Dietary intake data were analyzed by a research dietitian using Nutrition Data System for Research software version 2016, developed by the Nutrition Coordinating Center, University of Minnesota.

### 2.5 Physical activities

All participants were instructed to maintain their usual physical activity throughout the study. Each participant was interviewed at baseline and end of week 8 using the Physical Activity Recall Assessment for People with Spinal Cord Injury questionnaire (Ginis et al., 2005).

### 2.6 Metabolic function assessments

All testing procedures were performed at the University of Alabama at Birmingham. For the OGTT, each participant was given 5 min to consume a 75‐g oral glucose load after a 10–12 h overnight fast. Blood samples were collected immediately before and 10, 30, 60, 90, and 120 min after glucose ingestion for serum glucose and insulin measurement (Li et al., 2022). Serum glucose assays were performed on an automated analyzer (Sirrus analyzer; Stanbio Laboratory) and serum insulin and C‐peptide was measured using an immunofluorescent method with an AIA‐600 II analyzer (TOSOH Bioscience) as per the manufacturers' instructions. Whole‐body insulin sensitivity was calculated using the Matsuda index (a formula based on insulin and glucose values measured during the OGTT) (Matsuda & Defronzo, 1999). Glucose‐stimulated insulin secretion (GSIS) was calculated by dividing insulin increment by the glucose increment within 30 min of the OGTT (ΔInsulin_30_/ΔGlucose_30_) (Phillips et al., 1994). Disposition index, a pancreatic beta cell function index adjusted for insulin sensitivity, was calculated as GSIS * 1/fasting insulin. Incremental area under the curve (AUC) was calculated using the trapezoid rule for various indices. Hepatic insulin extraction was calculated using the equation [1−(incremental AUC_insulin_/incremental AUC_C‐Peptide_)]*100% (Tura et al., 2006). The fasting blood sample was used for lipid analysis (i.e., total cholesterol, triglyceride, high‐density lipoprotein cholesterol [HDL‐c], and low‐density lipoprotein cholesterol [LDL‐c]). Total cholesterol, HDL‐c, and triglyceride are measured with the Sirrus analyzer. LDL‐c levels are estimated using the formula LDL‐c = total cholesterol − HDL‐c − triglyceride/5 (Friedewald et al., 1972).

### 2.7 Dual‐energy x‐ray absorptiometry (DXA) scans

Total body fat, lean mass, and visceral fat mass were estimated by DXA scans (Hologic QDR‐4500W) using the enCORE software version 13.6 (G.E. Medical Systems) (Kaul et al., 2012). Percentage of fat mass and lean body mass were estimated using DXA results. For visceral fat, the android region is automatically defined, with the base of the region placed at the top of the iliac crest and the height set to 20% of the distance from the top of the iliac crest to the base of the skull (Kaul et al., 2012). Fat mass data from DXA are transformed from adipose tissue volume using a constant correction factor (0.94 g/cm^3^). This constant is generally consistent with the density of adipose tissue. Participants were scanned in light clothing while lying flat on their backs with their arms at their sides. Despite its wide use in assessing body composition in the general population, some limitations of DXA may introduce additional bias for individuals with SCI, such as interference of metal implants (Giangregorio & Webber, 2003) and bias when estimating body fat among individuals who are lean or obese (Laforgia et al., 2009). However, given that the main outcome of interest is the change value, we considered DXA a suitable method for accessing body composition changes among our participants.

### 2.8 Inflammation panel

Using the fasting sample collected for the OGTT, serum markers for chronic inflammation, including interleukins (IL) (i.e., IL‐6, IL‐8, IL‐10), and tumor necrosis factor‐α (TNF‐α) were analyzed in duplicate using the MesoScale Discovery Proinflammatory Kit. C‐reactive protein (CRP) was analyzed on a Sirrus Stanbio analyzer.

### 2.9 Statistical analysis

Descriptive statistics were calculated for all study variables of interest, with mean ± standard deviation reported for normally distributed continuous variables. Median and interquartile range (IQR) were reported for non‐normally distributed variables. Unpaired *t*‐tests and chi‐square tests were used to compare baseline group characteristics for continuous and categorical variables, respectively. A linear mixed‐effects model was built to compare dietary intake changes over time among participants in the control group. A linear mixed model analysis was constructed to test the effect of diet, time, and their interaction, with the participant as a random effect, and level of injury and sex as covariates. Pairwise post hoc comparisons were performed using the Tukey–Kramer multiple comparisons method to estimate within‐group changes. Statistical model assumptions were validated before data analysis. Statistical tests were two‐sided, and *p* < 0.05 was considered statistically significant. Statistical analyses were performed using the SAS, version 9.4 (SAS Institute, Inc.). Data presented are mean ± standard deviation unless otherwise stated. We intended to recruit 60 participants (30 for each group) to have 80% power to detect an effect size of 0.73 (Cohen's *d*) for between‐group differences in Matsuda index or the glucose concentration at minute 120 during the OGTT. However, due to COVID‐related interruptions, the final achieved sample size (*n* = 25) in this trial provides 80% power to detect an effect size of 1.18 at alpha level of 0.05. Thus, the results reported in this manuscript should be considered exploratory, rather than confirmatory.

## 3 RESULTS

### 3.1 Baseline characteristics

A total of 25 participants were included in this analysis. Participants did not differ in age, duration of injury, and distributions of demographic and injury‐related characteristics at baseline between groups (*p* > 0.05 for all) **(**Table 1 **)**. On average, participants from both groups consumed diets with ~16% energy from dietary protein, comparable to the average protein intake among Americans (Popp et al., 2019). In addition, their diets were high in dietary fat (>30% total energy intake), added sugar (>10% total energy intake), and low in dietary fiber (Table 2) compared to the 2020 U.S. dietary guidelines (U.S. Department of Health and Human Services, 2020) and the Dietary Reference Intakes established by the Institute of Medicine (Institute of Medicine, 2005).

**TABLE 1:** Baseline participants' characteristics

	Diet group	Control group	*p* *
Age, y*	40.6 ± 12.6	44.2 ± 10.9	0.54
Sex	8 M/3F	9 M/5F	0.65
Race/ethnicity	4 Non‐Hispanic Black	8 Non‐Hispanic Black	0.56
	6 Non‐Hispanic White	6 Non‐Hispanic White	
	1 Asian		
Level of injury	Cervical: 5	Cervical: 5	0.62
	Thoracic: 6	Thoracic: 9	
Completeness of injury	Complete: 8	Complete: 10	0.94
	Incomplete: 3	Incomplete: 4	
Duration of injury, y*	18.1 ± 13.7 (min: 3, max: 45)	18.1 ± 12.2 (min: 4, max: 53)	>0.99

*Note*: Data are mean ± SD.

* *p* values were derived from independent *t*‐tests or chi‐square tests.

**TABLE 2:** Dietary intake among study participants

(a) Nutrient composition of the baseline diet versus prescribed study diet among participants in the low‐carbohydrate/high protein group		
Nutrient	Baseline	Prescribed diet^a^
Calorie, kcal	1841 ± 533	2127 ± 397
Total fat, g	75.9 ± 19.8	75.9 ± 15.5
Total carbohydrate, g	223 ± 82	222 ± 39
Total protein, g	70.2 ± 20.5	151 ± 27
Saturated fat, g	23.4 ± 8.2	21.6 ± 4.7
Monounsaturated fat, g	27.9 ± 8.7	25.6 ± 3.9
Polyunsaturated fat, g	18.4 ± 4.9	19.6 ± 2.7
Total dietary fiber, g	13.8 ± 6.4	29.3 ± 5.6
Soluble dietary fiber, g	4.8 ± 2.3	20.9 ± 3
Insoluble dietary fiber, g	8.8 ± 4.5	6.1 ± 0.9
% Calories from fat	36 ± 3.9	31.3 ± 0.7
% Calories from carbohydrate	47.5 ± 5.7	40.3 ± 0.5
% Calories from protein	16.3 ± 4.1	28.4 ± 0.3
% Calories from saturated fat	11.1 ± 2.3	8.2 ± 2.9
Added sugars, g	77.7 ± 48.1	33 ± 11.7

(b) Dietary intake did not change over time among participants in the control group				
Nutrient	Baseline	Week 4	Week 8	*p*_time_ *
Calorie, kcal	1563 ± 667	1701 ± 588	1799 ± 556	0.27
Total fat, g	67.3 ± 29.3	76.8 ± 34.2	83.2 ± 30.3	0.13
Total carbohydrate, g	176 ± 94	186 ± 79	198 ± 84	0.21
Total protein, g	63.5 ± 29.1	67.3 ± 18.5	67.1 ± 15.8	0.71
Saturated fat, g	21.1 ± 7.4	23.2 ± 9.8	24.2 ± 8.1	0.32
Monounsaturated fat, g	24 ± 9.6	27 ± 12.1	29.4 ± 12.2	0.23
Polyunsaturated fat, g	18.8 ± 10.8	20.3 ± 11.1	23.3 ± 10.7	0.21
Total dietary fiber, g	10.2 ± 5.3	10.8 ± 5.5	11.5 ± 4.6	0.46
Soluble dietary fiber, g	4 ± 2.1	3.8 ± 2.1	4.6 ± 3.2	0.21
Insoluble dietary fiber, g	6.1 ± 3.8	6.9 ± 3.7	6.8 ± 1.8	0.60
% Calories from fat	38.8 ± 7.2	39.2 ± 9.2	40.6 ± 8.8	0.63
% Calories from carbohydrate	43.8 ± 7.8	43.1 ± 9.3	42.1 ± 10.4	0.76
% Calories from protein	16.9 ± 3	17 ± 4.1	16.9 ± 6.5	1
% Calories from saturated fat	12.1 ± 2.1	12 ± 2.8	12 ± 2.8	0.50
Added sugars, g	66.3 ± 49.5	65.6 ± 44.6	81 ± 53.8	0.24

^a^ Data are mean ± SD. Statistical comparison between baseline dietary intake and prescribed study diet was not conducted, due to differences in nutrient assessment (dietary recalls vs. average nutrient composition of the prescribed diet).

* Results from a linear mixed model with participant as a random effect.

### 3.2 Compliance

Based on the checklist provided, participants in the diet group, on average, consumed 94% (IQR: 91%–97%) of the food items provided. Based on the dietary recalls, participants in the control group did not change their diet significantly over time (Table 2). In addition, neither group changed their daily duration of physical activity over time (diet group: 136 ± 104 to 104 ± 107 min/d; control group: 111 ± 72 to 119 ± 65 min/d, *p* > 0.05 for both).

### 3.3 Body composition

**FIGURE 2:**
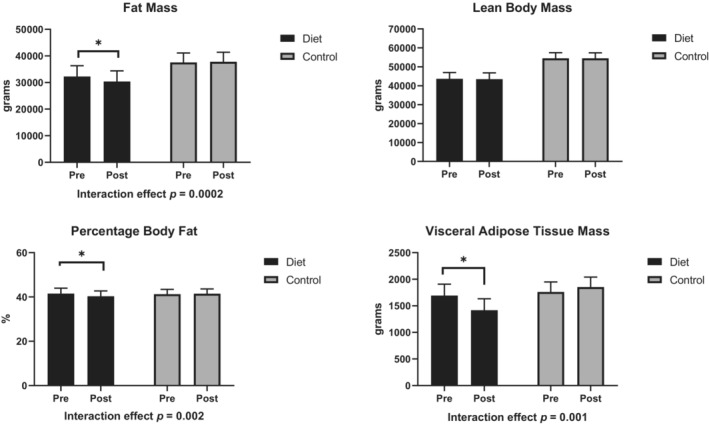
Changes in body composition in response to 8‐week interventions. Results are based on dual‐energy x‐ray absorptiometry (DXA) scans. Diet group: participants were provided with the low‐carbohydrate/high‐protein study diet; control group: participants consumed their usual diets. *p < 0.05 for within‐group changes. Data are least square means and standard errors derived from the linear mixed models.

Changes in body composition are shown in Figure 2 and Table S1. A significant effect was observed for diet‐by‐time interaction for fat mass, percentage body fat, and visceral adipose tissue mass (*p*_diet*time_ <0.05). Specifically, total body fat mass, percentage body fat, and visceral fat mass decreased by 5.9%, 2.9%, and 16.2% of their baseline values, respectively, in the LC/HP group (*p* < 0.05 for all within‐group changes). No significant changes in these outcomes were observed in the control group. Lean body mass did not change significantly over time in either group.

### 3.4 Glucose homeostasis

**FIGURE 3:**
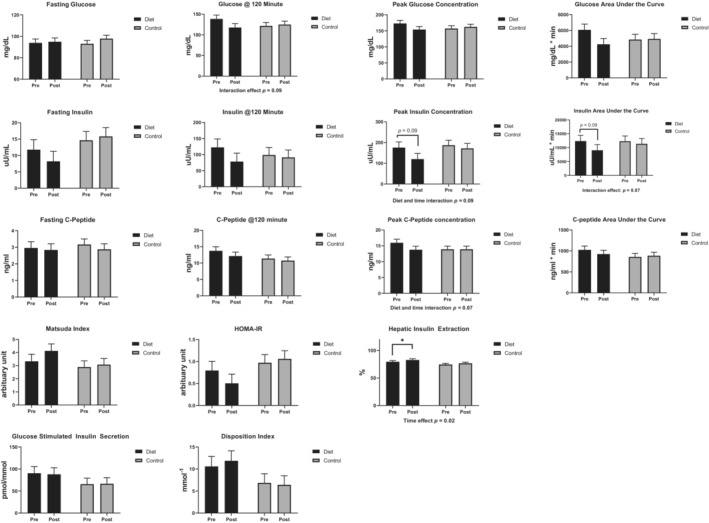
Changes in glucose metabolism among participants in response to the 8‐week interventions. Diet group: participants were provided with the low‐carbohydrate/high‐protein study diet; control group: participants consumed their usual diets. Results are based on a 2‐h oral glucose tolerance test with a 75‐g glucose load. Data are least square means and standard errors derived from the linear mixed models.

There were no significant main effects of time, diet, and their interaction on the fasting concentrations of glucose and insulin, insulin concentrations at 120‐min during the OGTT, glucose AUC, peak glucose concentrations during the OGTT, Matsuda index, GSIS, and disposition index. A trend for interaction effect was observed for glucose concentration at minute 120 during the OGTT (*p*_diet*time_ = 0.09) and peak C‐peptide concentrations during the OGTT (*p*_diet*time_ = 0.07), where their concentrations decreased quantitatively among participants in the LC/HP group. Regardless of group assignment, insulin AUC (*p* = 0.04) and peak insulin concentrations (*p* = 0.03) decreased over time, and HIE (*p* = 0.03) increased over time. Though the diet‐by‐time interaction effect was not significant, the changes in insulin and HIE seem to be driven by those observed in the LC/HP group (Figure 3 and Table S1).

### 3.5 Lipid profile

**FIGURE 4:**
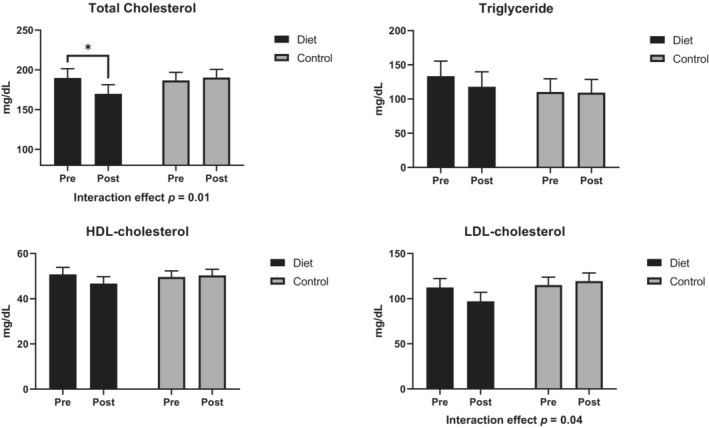
Changes in fasting blood lipid profile in response to the 8‐week interventions. Diet group: participants were provided with the low‐carbohydrate/high‐protein study diet; control group: participants consumed their usual diets. *p < 0.05 for within‐group changes. Data are least square means and standard errors derived from the linear mixed models.

Participants in the LC/HP diet group had favorable changes in lipid profiles compared to the control group (Figure 4 and Table S1). A significant effect for diet and time interaction was observed for total cholesterol (*p* = 0.02) and LDL‐c (*p* = 0.04). Among participants in the diet group, fasting total cholesterol decreased by −20.1 ± 28.0 mg/dl (*p* < 0.05), while the control group had minimal changes. LDL‐c quantitively decreased by 13.9 ± 26.8 mg/dl in the LC/HP group. No effects of diet, time, and their interaction were observed for triglycerides and HDL.

### 3.6 Inflammation panel

Several markers of chronic inflammation, including CRP, IL‐6, IL‐8, IL‐10, and TNF‐alpha, did not change differently between groups over time (Table 3 and Table S1).

**TABLE 3:** Changes in markers of chronic inflammation in response to the 8‐week dietary interventions

	Control group		Low‐carbohydrate/High‐protein group		*p*_diet_	*p*_time_	*p*_interaction_
	Baseline	Week 8	Baseline	Week 8			
C‐reactive protein, mg/L	4.9 ± 4.1	4.4 ± 2.2	5.5 ± 6.9	6.1 ± 7.4	0.81	0.87	0.91
IL‐10, pg/ml	0.3 ± 0.1	0.3 ± 0.1	0.3 ± 0.2	0.4 ± 0.3	0.39	0.28	0.21
IL‐6, pg/ml	2.0 ± 1.5	1.7 ± 1.1	1.3 ± 0.7	1.6 ± 0.9	0.22	0.97	0.28
IL‐8, pg/ml	10.5 ± 3.1	12 ± 4	12.3 ± 4.5	14 ± 4.9	0.23	0.11	0.71
TNF‐α, pg/ml	2.8 ± 1.6	2.7 ± 1.4	2.2 ± 0.7	2.2 ± 0.7	0.12	0.64	0.68

*Note*: Data are mean ± SD. Diet group: participants were provided with the low‐carbohydrate/high‐protein study diet; control group: participants consumed their usual diets.

### 3.7 Important harms or unintended effects in each group

No participant from either group reported any adverse events during the study.

## 4 DISCUSSION

Given the pathophysiological changes accompanied by SCI, such as muscle loss, increased adiposity, and impaired metabolism, unique nutritional considerations may be necessary to ameliorate and prevent the development of metabolic disorders (Bigford & Nash, 2017). Existing research conducted in the general population supports that diets with a higher protein content may counteract these SCI‐associated changes. Consistent with results observed in the general population (Leidy, 2014; Weigle et al., 2005) and our hypothesis, our data support that an LC/HP diet with healthy dietary components can improve body composition and blood lipid profile in individuals with SCI.

In the current study, participants in the LC/HP group had reduced body fat and visceral adiposity after the intervention, consistent with a meta‐analysis of isocaloric diets with high versus normal/low protein content (Leidy, 2014; Santesso et al., 2012). Even though the study was not designed to delineate the mechanism of the LC/HP diet, research in the general population suggests that the fat mass loss may be related to several unique properties of dietary protein, compared to fat and carbohydrate. First, a higher dietary protein intake can increase whole‐body energy expenditure, including resting energy expenditure and the thermic effect of feeding (Halton & Hu, 2004; Westerterp‐Plantenga et al., 2009). As a result, consuming higher protein may partly compensate for the reduced energy expenditure in SCI. Second, when meals with a higher carbohydrate content are consumed, elevated insulin, which has anti‐lipolytic properties (Arner, 2005), promotes de novo lipogenesis from digested and absorbed simple sugars (e.g., glucose, fructose). Thus, partly replacing dietary carbohydrate with protein can reduce fat accumulation by limiting substrate availability for lipogenesis. Notably, total lean body mass was preserved despite of fat mass loss among participants in the LC/HP group. Individuals with SCI experience significant muscle loss due to the lack of muscle innervation (Castro et al., 1999; Clark & Findlay, 2017; Gorgey et al., 2014). Given the essential role of muscle in energy metabolism and glucose metabolism (Evans et al., 2019; Zurlo et al., 1990), preserving lean body mass should be a main goal for any dietary and lifestyle regimens for this population. The current study supports that an LC/HP diet may be adopted for improving body composition in individuals with SCI without calorie restriction.

In addition, we observed favorable changes in blood lipid profile among participants in the LC/HP group. Cholesterol concentrations decreased from 189.9 mg/dl, the higher end of the normal range at baseline, to 169.8 mg/dl after the intervention. Similarly, LDL‐c concentration was reduced to an optimal level (110 to 99 mg/dl) after the dietary intervention. This improvement may result from reduced total carbohydrate intake and changes in the type and quality of carbohydrates consumed, which collectively affect the meals' glycemic load (GL). The GL is estimated by multiplying the quality of carbohydrates in a given food by the amount of carbohydrates in a serving of that food. The study diet contained 40% energy from dietary carbohydrates. In comparison, participants consumed an average of 48% energy from dietary carbohydrate at baseline and had an excessive added sugar intake. The 2020 dietary guideline for Americans recommends limiting added sugar to less than 10% of calories per day (U.S. Department of Health and Human Services, 2020). On average, participants in the diet group consumed ~17% of calories from added sugars at baseline. Thus, the GL of the study diet was substantially lower than that of their baseline diet (study diet: average GL = 85, baseline diet: average GL = 128). Meals with elevated GL may cause increased postprandial glucose and insulin responses that promote liver de novo lipogenesis (i.e., production of free fatty acids), as well as the production of cholesterol and triglyceride‐containing lipoproteins (e.g., LDL‐c) (Sanders & Griffin, 2016). In support of this, several meta‐analyses of clinical trial studies showed that diets with lower GL can effectively improve blood lipid profiles (Chiavaroli et al., 2021; Goff et al., 2013).

Our data also suggest a potential benefit of the LC/HP diet on glycemic control. We observed a trend for diet and time interaction effect (*p* = 0.09), where glucose concentration at minute 120 during the OGTT decreased from 138.5 ± 11.2 to 117.7 ± 8.3 mg/d among participants in the diet group. The achieved effect size for the between‐group difference (Cohen's *d* = 0.70) is close to the expected effect size of 0.73 in our initial power calculation. Thus, the study diet may benefit glycemic control, but the current sample size is insufficient to reject the null hypothesis. Nevertheless, such a decrease is clinically meaningful. Upon closer examination of individual responses, at baseline, 5 participants in the LC/HP and 4 participants in the control group had pre‐diabetes. After the intervention, four out of five participants in the LC/HP group had remission of their pre‐diabetes and achieved normal glucose tolerance. In contrast, none of the four participants in the control group had remission of their pre‐diabetes. Furthermore, given that pancreatic beta bell function did not change over time based on the GSIS and DI data, the trend for reduced insulin responses (i.e., insulin AUC and peak insulin/C‐peptide concentrations) indicates improved insulin sensitivity. The increased HIE levels further support the potential benefit of the LC/HP diet on glucose metabolism. Previous studies showed that HIE is reduced in those with pre‐diabetes (Bonora et al., 1983), and HIE is highly correlated with insulin sensitivity (Utzschneider et al., 2019). Despite the well‐accepted favorable impact of a higher protein intake on weight control and metabolic health (Gannon et al., 2003; Leidy et al., 2015), the direct impact of a dietary protein on insulin sensitivity remains inconclusive (Krebs et al., 2002). Research showed that higher protein intake could either reduce (Te Morenga et al., 2017) or improve insulin sensitivity (Yu et al., 2020). In the current study, given that the study diet partially replaced participants' refined grains and sugar intake with lean proteins and fiber‐rich carbohydrate, the higher carbohydrate quality may have contributed to the improvements in glucose metabolism. Refined grains cause increased postprandial glucose and insulin concentrations, which are causally related to the development of obesity, type 2 diabetes, and cardiovascular diseases (Gross et al., 2004; Ludwig, 2002).

Consistent with previous observations, at the study baseline, participants with SCI had elevated markers of chronic inflammation (Allison & Ditor, 2015), such as CRP (~5.2 mg/L, normal range < 2 mg/L). Research showed that chronic inflammation associated with obesity impairs insulin signaling pathways and lipid metabolism (Monteiro & Azevedo, 2010). However, despite the improvement in body composition, lipid profile, and insulin sensitivity, we observed no changes in several serum biomarkers for chronic inflammation. A recent meta‐analysis suggested that for every 1 kg of weight loss, the average reduction of CRP is 0.13 mg/L (Selvin et al., 2007). Thus, given the limited weight loss (~2 kg) among participants in the LC/HP group, improvements in these inflammatory markers may not be apparent among these participants.

Dietary intervention is one of the key strategies for weight loss, which has been considered the quintessential step to achieving health benefits. However, accumulating evidence supports that certain dietary interventions can improve metabolic health without the need for substantial weight loss (Meslier et al., 2020; Nuttall et al., 2008). In our study, the overall degree of weight loss was small (~2 kg), and we found no significant correlations between the changes in body composition with the health outcomes (lipid, glucose, and inflammation, correlation data not shown). However, it remains unknown whether the LC/HP‐induced improvements were weight loss‐ and fat loss‐dependent among our participants, as with the current sample size, we are only equipped to detect correlations of 0.7 or greater based on a two‐tailed Type I error rate of 0.05 and 80% power.

### 4.1 Study limitations

Our study has several limitations. First, participants in the control group consumed their usual diet, which had a normal protein content (~16%) as well as low diet quality (e.g., low fruits and vegetables, high refined grain and added sugar, low fiber). Thus, it is unclear whether the improvements in the study outcomes among participants in the LC/HP group were due to the differences in macronutrient composition or a combination of both macronutrient changes and a healthier dietary pattern. Second, due to the small sample size, it was not feasible to evaluate whether the impact of the diet differed for participants in different age groups, sexes, levels of injury, completeness of injury, or duration of the injury, as well as identify potential responders vs. non‐responders to the dietary intervention. As a result, the study result may not be generalizable to all individuals with SCI.

## 5 CONCLUSIONS

Our exploratory analysis supports a favorable change in the body composition and blood lipid profile after consuming a healthy LC/HP diet for 8 weeks in individuals with chronic SCI. The impact of the study diet on glucose metabolism and inflammation is inconclusive and warrants future evaluations. Nevertheless, our results underscored the importance of dietary modifications for metabolic health in SCI. Long‐term safety and efficacy diet trials for preventing and managing metabolic disorders in individuals with SCI are warranted.

## AUTHOR CONTRIBUTIONS

The authors confirm contribution to the paper as follows: study conception and design: Jia Li, Ceren Yarar‐Fisher, Amie McLain, and Barbara Gower, participant recruitment and data collection: Jia Li, analysis and interpretation of results: Jia Li and Ceren Yarar‐Fisher; draft manuscript preparation: Jia Li, Ceren Yarar‐Fisher, Amie McLain, and Barbara Gower. All authors reviewed the results and approved the final version of the manuscript.

## FUNDING INFORMATION

This work was supported by the National Institute on Disability, Independent Living, and Rehabilitation Research (NIDILRR UAB Spinal Cord Injury Model Systems Grant: NIDILRR‐90SI5019) and the National Center for Advancing Translational Sciences of the National Institutes of Health under award number UL1TR003096.

## CONFLICT OF INTEREST

The authors declare no competing interests during the course of this research.

## ETHICS STATEMENT

The University of Alabama at Birmingham institutional review board approved the study. We certify that all applicable institutional and governmental regulations concerning the ethical use of human volunteers/animals were followed.

## Supporting information


Table S1
