# Supplementary material for: Effects of a low‐carbohydrate/high‐protein diet on metabolic health in individuals with chronic spinal cord injury: An exploratory analysis of results from a randomized controlled trial
Source: Physiol Rep. 2022 Nov 21;10(22):e15501. doi: 10.14814/phy2.15501 (PMC9812250; doi:10.14814/phy2.15501)
Supplement: Supplementary file 1 — Table S1 [file PHY2-10-e15501-s001.docx]

|  | **Control Group** | | | **Low-Carbohydrate/High-Protein Diet Group** | | | **Linear Mixed Model Results** | | |
| --- | --- | --- | --- | --- | --- | --- | --- | --- | --- |
|  | **Post-Pre change** | **P*** | **Effect size** | **Post-Pre change** | **P*** | **Effect size** | **P^#^_Trial_** | **P^#^_Time_** | **P^#^_Trial*Time_** |
| **Cholesterol, mg/dL** | 3.7 ± 21.6 | 0.94 | 0.17 | -20.1 ± 28.0 | **0.03** | 0.72 | 0.54 | 0.11 | **0.02** |
| **Triglyceride, mg/dL** | -1.1 ± 24.6 | 1 | 0.04 | -15.5 ± 68.7 | 0.88 | 0.23 | 0.55 | 0.41 | 0.47 |
| **HDL-Cholesterol, mg/dL** | 0.7 ± 4.9 | 0.98 | 0.14 | -4.1 ± 9.0 | 0.24 | 0.45 | 0.75 | 0.24 | 0.10 |
| **LDL-Cholesterol, mg/dL** | 3.1 ± 14.5 | 0.94 | 0.34 | -13.9 ± 27.5 | 0.22 | 0.53 | 0.31 | 0.24 | **0.04** |
|  |  |  |  |  |  |  |  |  |  |
| **C-reactive protein mg/L** | -0.5 ± 3.3 | 0.99 | 0.14 | 0.6 ± 8.6 | 0.99 | 0.07 | 0.64 | 0.95 | 0.66 |
| **Interleukin 10, pg/ml** | 0.0 ± 0.1 | 0.99 | 0.05 | 0.1 ± 0.2 | 0.83 | 0.39 | 0.4 | 0.65 | 0.42 |
| **Interleukin 6, pg/ml** | -0.2 ± 0.9 | 0.79 | 0.25 | 0.2 ± 0.9 | 0.83 | 0.27 | 0.26 | 0.98 | 0.22 |
| **Interleukin 8, pg/ml** | 1.4 ± 3.7 | 0.59 | 0.39 | 1.8 ± 2.1 | 0.48 | 0.84 | 0.3 | 0.07 | 0.81 |
| **Tumour Necrosis Factor alpha, pg/ml** | -0.1 ± 0.4 | 0.99 | 0.24 | 0.04 ± 0.57 | 0.99 | 0.07 | 0.15 | 0.96 | 0.71 |
|  |  |  |  |  |  |  |  |  |  |
| **Fat-free mass, g** | -17 ± 1671 | 1 | 0.01 | -173 ± 1649 | 0.99 | 0.10 | 0.02 | 0.78 | 0.82 |
| **Fat mass, g** | 264 ± 878 | 0.85 | 0.30 | -1890 ± 1582 | **0.00** | 1.19 | 0.23 | 0.00 | **0.00** |
| **Lean mass, g** | -71 ± 1654 | 1 | 0.04 | -81 ± 1610 | 1 | 0.05 | 0.02 | 0.82 | 0.99 |
| **Body weight, g** | 247 ± 1778 | 0.98 | 0.14 | -2063 ± 2724 | **0.03** | 0.76 | 0.05 | 0.06 | **0.02** |
| **Total body fat, %** | 0.15 ± 0.94 | 0.93 | 0.16 | -1.22 ± 1.0 | **0** | 1.19 | 0.88 | 0.01 | **0** |
| **Visceral adipose tissue, g** | 91 ± 205 | 0.48 | 0.45 | -274 ± 255 | **0.01** | 1.08 | 0.37 | 0.07 | **0** |
|  |  |  |  |  |  |  |  |  |  |
| **Fasting glucose, mg/dL** | 4.7 ± 10.7 | 0.52 | 0.44 | 1.0 ± 14.9 | 0.99 | 0.07 | 0.79 | 0.27 | 0.49 |
| **Glucose_OGTT 120-minute_, mg/dL** | 3.0 ± 31.3 | 0.99 | 0.09 | -20.7 ± 37.0 | 0.21 | 0.56 | 0.63 | 0.21 | **0.09** |
| **Incremental glucose area under the curve, mg/dL*min** | 901 ± 5790 | 0.96 | 0.03 | -1823± 3806 | 0.40 | 0.48 | 0.75 | 0.15 | 0.18 |
| **Peak glucose during the OGTT, mg/dL** | 4.9 ± 32.0 | 0.96 | 0.15 | -19.2 ± 46.2 | 0.38 | 0.42 | 0.7 | 0.37 | 0.14 |
| **Time to peak for glucose, min** | 11.5 ± 33.6 | 0.71 | 0.34 | -5.5 ± 44.1 | 0.97 | 0.12 | 0.65 | 0.71 | 0.30 |
|  |  |  |  |  |  |  |  |  |  |
| **Fasting Insulin, uU/mL** | 1.2 ± 14.2 | 0.98 | 0.08 | -3.6 ± 6.1 | 0.73 | 0.58 | 0.12 | 0.61 | 0.31 |
| **Insulin_OGTT 120-minute_, uU/mL** | -7.5 ± 81.4 | 0.99 | 0.09 | -44.2 ± 104.2 | 0.40 | 0.42 | 0.85 | 0.18 | 0.33 |
| **Incremental insulin area under the curve, uU/mL*min** | -2380 ± 8213 | 0.83 | 0.95 | -3301 ± 6087 | **0.09** | 0.54 | 0.47 | **0.04** | 0.16 |
| **Peak insulin during the OGTT, uU/mL** | -15.5 ± 65.3 | 0.86 | 0.24 | -55.6 ± 83.7 | **0.09** | 0.66 | 0.34 | **0.03** | 0.19 |
| **Time to peak for insulin, min** | 9.2 ± 46.5 | 0.88 | 0.2 | -8.2 ± 42.6 | 0.93 | 0.19 | 0.57 | 0.95 | 0.35 |
|  |  |  |  |  |  |  |  |  |  |
| **Fasting C-peptide, ng/ml** | -0.3 ± 1.4 | 0.87 | 0.20 | -0.1 ± 1.4 | 0.99 | 0.08 | 0.76 | 0.48 | 0.77 |
| **Cpep_OGTT 120-minute_, ng/ml** | -0.6 ± 4.3 | 0.96 | 0.14 | -1.6 ± 4.8 | 0.64 | 0.34 | 0.18 | 0.23 | 0.59 |
| **Incremental C-peptide area under the curve, ng/ml*min** | -29 ± 238 | 0.97 | 0.12 | -100 ± 220 | 0.48 | 0.46 | 0.35 | 0.46 | 0.18 |
| **Peak C-peptide during the OGTT, ng/ml** | 0 ± 2.2 | 1 | 0.00 | -2.12 ± 3.6 | **0.08** | 0.61 | 0.48 | 0.07 | **0.07** |
| **Time to peak for C-peptide, min** | -2.3 ± 46.6 | 1 | 0.05 | 0.0 ± 42.4 | 1 | 0.00 | 0.77 | 0.9 | 0.90 |
|  |  |  |  |  |  |  |  |  |  |
| **Glucose stimulated insulin secretion, pmol/mmol** | 0.9 ± 33.1 | 1 | 0.03 | -2.5 ± 52.3 | 1 | 0.05 | 0.2 | 0.93 | 0.85 |
| **Disposition index, /mmol** | -0.5 ± 6.5 | 1 | 0.07 | 1.3 ± 8.0 | 0.94 | 0.16 | 0.09 | 0.78 | 0.57 |
| **Matsuda Index** | 0.2 ± 1.6 | 0.96 | 0.12 | 0.7 ± 1.2 | 0.53 | 0.62 | 0.13 | 0.49 | 0.20 |
| **Hepatic insulin extraction, %** | 2.1 ± 5.7 | 0.91 | 0.21 | 3.3 ± 6.1 | **0.05** | 0.55 | 0.07 | **0.03** | 0.61 |
| **Homeostatic Model Assessment for Insulin Resistance** | 0.09 ± 0.8 | 0.96 | 0.11 | -0.29 ± 0.57 | 0.53 | 0.51 | 0.13 | 0.49 | 0.20 |

Data are Mean ± SD. *P value for within group changes overtime based on post hoc comparisons under the linear mixed effect model. #P values are obtained from linear mixed effect model.
